# Correlations Between the Expression of Stromal Cell Activation Related Biomarkers, L-NGFR, Phospho-ERK1-2 and CXCL12, and Primary Myelofibrosis Progression

**DOI:** 10.3389/pore.2022.1610217

**Published:** 2022-03-14

**Authors:** Tamas Szekely, Tibor Krenacs, Mate Elod Maros, Csaba Bodor, Viktoria Daubner, Annamaria Csizmadia, Brigitta Vrabely, Botond Timar

**Affiliations:** ^1^ First Department of Pathology and Experimental Cancer Research, Semmelweis University, Budapest, Hungary; ^2^ Department of Biomedical Informatics, Center for Preventive Medicine and Digital Health, Mannheim, Germany; ^3^ Department of Neuroradiology, Medical Faculty Mannheim, University of Heidelberg, Mannheim, Germany; ^4^ HCEMM-SE Molecular Oncohematology Research Group, Budapest, Hungary; ^5^ 3DHISTECH Ltd., Budapest, Hungary; ^6^ Department of Pathology, Sandor Peterfy Street Hospital and Clinic, Budapest, Hungary

**Keywords:** primary myelofibrosis progression, -stromal cell activation, -L-NGFR/CD271, -CXCL12, -phospho-ERK1-2, -connexin 43 channels

## Abstract

In myelofibrosis, pathologically enhanced extracellular matrix production due to aberrant cytokine signalling and *clonal megakaryocyte functions* result(*s*) in impaired hemopoiesis. Disease progression is still determined by detecting reticulin and collagen fibrosis with Gomori’s silver impregnation. Here, we tested whether the expression growth related biomarkers L-NGFR/CD271, phospho-ERK1-2 and CXCL12 can be linked to the functional activation of bone marrow stromal cells during primary myelofibrosis progression. Immunoscores for all tested biomarkers showed varying strength of positive statistical correlation with the silver impregnation based myelofibrosis grades. The intimate relationship between spindle shaped stromal cells positive for all three markers and aberrant megakaryocytes was likely to reflect their functional cooperation. L-NGFR reaction was restricted to bone marrow stromal cells and revealed the whole length of their processes. Also, L-NGFR positive cells showed the most intersections, the best statistical correlations with myelofibrosis grades and the strongest interrater agreements. CXCL12 reaction highlighted stromal cell bodies and a weak extracellular staining in line with its constitutive release. Phospho-ERK1-2 reaction showed a similar pattern to CXCL12 in stromal cells with an additional nuclear staining in agreement with its role as a transcription factor. Both *p*-ERK1-2 and CXCL12 were also expressed at a moderate level in sinus endothelial cells. Connexin 43 gap junction communication channels, known to be required for CXCL12 release to maintain stem cell niche, were also expressed progressively in the myelofibrotic stromal network as a support of compartmental functions. Our results suggest that, diverse growth related pathways are activated in the functionally coupled bone marrow stromal cells during myelofibrosis progression. L-NGFR expression can be a useful biological marker of stromal cell activation which deserves diagnostic consideration for complementing Gomori’s silver impregnation.

## Introduction

Primary myelofibrosis (PMF) belongs to a group of Philadelphia chromosome (BCR-ABL1)-negative myeloproliferative neoplasms (MPN) of the multipotent hematopoietic stem cells, also including polycythaemia vera (PV) and essential thrombocythemia (ET) [1]. These malignancies are characterised by clonal proliferation of the myeloid lineages accompanied by progressive stromal cell activation, extracellular matrix production and impeded hemopoesis [2]. Myelofibrosis is an adverse prognostic factor in MPNs, which is mainly driven by impaired megakaryocyte functions resulting in the elevated expression of inflammatory cytokines, transforming growth factor-β (TGF-β), platelet derived growth factor (PDGF), as well as the aberrant JAK-STAT signaling as a result of JAK2V617F, MPL515 L/K or CALR mutations [3]. Myelofibrosis grading is still based on Gomori’s silver staining, which reveals reticular and collagen fibers proportional with disease progression [4]. However, standardisation of the selective silver impregnation of these matrix components is challenging due to preanalytical and staining variables [5]. Here we studied, the growth related biomarkers L-NGFR, phospho-ERK1-2 and CXCL12 in primary myelofibrosis, which were predominantly expressed in bone marrow stromal cells and potentially involved in the promotion of fibroblast activation, responsible for accelerated and pathognomic matrix production during myelofibrosis. Correlations between the expression of these makers and myelofibrosis grades were tested to see if they could support reticulin silver impregnation based prognostic decisions on a biological basis.

Low affinity nerve growth factor receptor (L-NGFR; CD271, or p75 neurotrophin receptor - p75NTR), an unusual member of the tumor necrosis factor family of receptors, has long been detected in the bone marrow (reticular) stromal cell network [6]. L-NGFR has been involved in the regulation of neuronal survival and apoptosis but it can also act as a tyrosine kinase co-receptor for the anti-apoptotic tropomyosin receptor kinase A (TrkA) to enhance MAPK pathway activation, culminating in ERK1-2 phosphorylation and nuclear translocation [7, 8]. Both L-NGFR expression and NGF-TrkA signaling can support bone and bone marrow mesenchymal stem cell (MSc) development [9, 10]. In addition, TrkA upregulation can enhance the survival and regenerative capacity of bone marrow stromal stem cells through upregulation of the Erk/Bcl-2 pathway [11]. In myelofibrotic bone marrow MAPK activation can also be induced through receptor tyrosine kinase signaling i.e. PGDFR by PDGF overproduced by pathological megakaryocytes, as one of the major driving forces of fibroblast activation [12]. In line with this, PDGFR-β expression in the stromal network showed close correlation with the silver impregnation based myelofibrosis grades [13-15].

The chemokine CXCL12 (stromal cell-derived factor 1 - SDF1, or C-X-C motif chemokine 12) produced by stromal and endothelial cells and osteoblasts, plays an important role in maintaining stem cell quiescence by safeguarding both the perivascular or endosteal bone marrow niches [16]. Its receptor CXCR4 is known to be expressed in CD34+/c-kit + hemopoetic progenitors and leukemic blasts. Mesenchymal stromal cells can resist in radiation-induced cell death and the CXCL12 (SDF1)‐CXCR4 signaling in the stromal microenvironment promotes niche regeneration during stem cell transplantation [17]. Bone marrow stromal cells, as a dynamic syncytium, communicate via Cx43 (and Cx45) gap junctions, which are required for proper CXC12 secretion and hematopoietic stem cell homeostasis [18]. Accordingly, inhibition of gap junctions impaired CXCL12 secretion and the homing of CD34 ^+^ bone marrow progenitors. We have earlier demonstrated the upregulation of Cx43 gap junctions in leukemic tumor samples, characterized by an increased stromal/hematopoietic cell ratio [19].

The CXCL12/CXCR4 pathway can be induced by oncogenic JAK2, which frequently suffers activating mutations in primary myelofibrosis, and JAK2 inhibition can reduce the chemotaxis of hematopoietic cells isolated from primary myelofibrosis [20]. A subpopulation of leukemic cells with stem cell-like features can maintain tumor growth by escaping antitumor therapies and repopulating the tumor [21]. Myelofibrosis, featured by increased stromal cell activation, may also involve CXCL12 upregulation, which can contribute to protecting leukemic stem cells [22].

In this study, using immunohistochemistry, we tested the expression of L-NGFR, pER1-2 and CXCL12 proteins, which were primarily expressed in stromal cells in bone marrow biopsies of primary myelofibrosis. We also studied Cx43 communication channel protein expression in the myelofibrotic stroma. All these markers are potentially involved in the promotion of growth and activation of bone marrow stromal network to produce excess matrix proteins including reticular and collagen fibers, which determine myelofibrosis progression. Therefore, we examined for their correlations with stromal fibrosis in myelofibrosis in association with Gomori’s-silver impregnation based tumor grades.

## Materials and Methods

### Bone Marrow Biopsy Samples Tested

Our cohort included Jamshidi biopsies of patients diagnosed with primary myelofibrosis at the first Department of Pathology, Semmelweis University between 2016 and 2021. Samples were fixed for 10–16 h in Schaefer’s fixative (4% neutral buffered formaldehyde containing methanol and glucose), then decalcified overnight in 10% EDTA-Na_2_ (ethylenediaminetetraacetic acid disodium salt) and embedded routinely into paraffin wax. Having excluded some fragmented, or non-representative samples, overall 60 were selected. All cases were reevaluated and the grades confirmed by an expert hematopathologist based on the WHO 2016 criteria. Thirty six cases were considered as grade 3 (MF-3) (22 males, 14 females; median age 66.3 years; range 45–86), 18 cases as grade 2 (MF-2) (5 males, 13 females; median age 56.2 years; range 31–76) and 6 cases were diagnosed as prefibrotic myelofibrosis with grade 1 (MF-1) fibrosis (3 males, three females; median age 54.1 years; range 29–86). Forty eight patients (80%) carried either the JAK2 V617F mutation (43 out of 60; 4 MF-1, 13 MF-2 and 26 MF-3 cases; 71,7%) or CALR mutations (5 out of 60; 2 MF-2 and 3 MF-3 cases; 8.3%). Four out of five patients harboured type 1 and 1 patient had a type 2 CALR mutation. Twelve patients (20%) were double negative for the JAK2 and CALR analysis. The study was conducted in accordance with the Helsinki Declaration, and the application for ethical approval has been submitted.

Myelofibrosis grading was based on routine Gomori’s reticular fiber staining, which can also reveal excess of collagen fibers (collagen fibrosis). Briefly, rehydrated slides were oxidized using 1% potassium permanganate for 2 min followed by decoloration in 2% potassium metabisulfite for 1 min, then curing was done in 2% ferric ammonium sulphate solution for 1 min and silver impregnation in 10% silver nitrate containing 2% potassium hydroxide also for 1 min. Staining was finished with sequential treatments in 10% formalin for 5 min, then in 0.02% gold chloride for 10 s, 2% potassium metabisulfite for 1 min and finally in 1% sodium thiosulphate also for 1 min. The sections were washed in distilled water between incubation steps and mounted after dehydration.

Our diagnostic reticulin grading followed the European Consensus set up for bone marrow fibrosis [23]: MF-0, only perivascular reticulin fragments with no intersections; MF-1, focal, loose reticulin network with mainly perivascular intersections; MF-2, paratrabecular or central deposition of dense reticulin with regular intersections and occasional collagen bundles; MF-3: diffuse and dense intersecting reticulin network and collagen bundles with osteosclerosis.

### Immunohistochemistry

For immunohistochemistry 3 µm think sections mounted on adhesive glass slides were heat activated for >2 h at 62 °C and dewaxed after reaching room temperature. Immunostaining, except for CXCL12, was done using the Ventana Benchmark Ultra automated system (Roche Diagnostics, Tucson, AR) including antigen retrieval for 40 min in the high pH CC1 buffer, incubation with the primary antibodies for 60 min and then with the Ultraview detection system for 40 min, and visualization using DAB/hydrogen peroxide development. CXCL12 was detected with manual immunostaining after TRIS-EDTA retrieval (pH 9) for 40 min, using the same incubation times as in the automated system with peroxidase conjugated Histols micropolymer (Histopathology Kft, Pécs) and revealed with DAB Quanto (TA-060-PHDX, Thermo Sci. Runcon, United Kingdom) kit. All immunoreactions were completed with nuclear counterstaining using hematoxylin.

Primary antibodies used in this study were monoclonal mouse anti-human p75 low affinity nerve growth factor receptor (L-NGFR, 1:100, clone: 7F10, Leica-NovoCastra, Newcastle, United Kingdom), CXCL12/SDF-1⟨ (1:50, clone:#79018, R&D, Minneapolis, MN, United States), rabbit anti-phospho-ERK1-2 (p44/42; Thr202/Tyr204; 1:100, clone:20G11, Cell Signaling, Danvers, MA, United States), as well as rabbit polyclonal connexin43 (Cx43, 1:100, #3512, Cell Signaling).

In addition, the rabbit anti-Cx43 (1:100) and the mouse anti-L-NGFR (1:100) primary antibodies were simultaneously combined in 10 cases of myelofibrosis for double immunofluorescence, and detected using a mixture of Alexa Fluor 488 (green, 1:200) or Alexa Fluor 546 (red, 1:200) conjugated goat anti-mouse/or -rabbit (IgG H + L) antibodies (both Invitrogen/-Life Technologies, Eugene, OR, United States), for 90 min incubations at each sequence. Cell nuclei were revealed in blue using the DNA staining Hoechst (bisbenzimide,1:500; Sigma-Aldrich, St. Louis, MO, United States) fluorescing dye for 1 min, and finally the sections were coverslipped with fluorescent mounting medium (Dako).

### Scoring and Image Analysis on Digital Slides

All immunostained sections were digitalized using the Pannoramic Scan system (3DHistech Ltd, Budapest) and scored either by visual analysis in the Pannoramic Viewer software or by using the HistoQuant semi-automated scoring program of the QuantCenter software package (all 3DHistech) for L-NGFR and Cx43 double stained fluorescence samples. At visual scoring we considered DAB reactions in elongated cells and their processes positive for the tested marker immunoreactions. The whole sections were taken into account but the final scores were determined by the areas of the highest density and intensity of positive immunreactions. Each marker reaction was assessed only within its own class to reveal potential correlations between its relative expression scores and Gomori’s silver impregnation based grading. Three categories, similar to silver grading, were used; score 1: scarce and weak positive; score 2: moderate/medium density and/or positive; and score 3: high density and intensity of the defined positive structures representing elongated cells and cell processes.

Double immunofluorescence (NGFR plus Cx43) reactions of 10 cases of myelofibrosis were studied using semi-automated image analysis as described before [24]. Immunostained slides were digitalized at separated color channels (R, G and B for cell nuclei) through five layers at each field of views (FOVs) and analyzed at extended focus using the HistoQuant program (3DHistech). Five to eight digital annotations representing the given samples were made in each slide, making up 67 areas (20 x MF-3, 20 x MF-2 and 20 x MF-1) to be tested. The automated semiquantitative image analysis was based on the measurement of immunopositive area fractions within the selected annotations. Saturated immunofluorescent signals at each relevant channel (red or green) were highlighted by image segmentation and considered to be positive. The segmented areas were measures in µm2 and the results were extrapolated and standardized to 1 mm^2^, and the correlations between the expression of the two markers were determined.

### Statistical Analyses

All analyses were performed with the R statistics program (v.4.1.0, R Core Team 2021, Vienna Austria; RStudio IDE v. April 1, Boston, MA, United States). Non-normally distributed variables were displayed as median, range and interquartile range (IQR). Categorical variables were reported as proportions. The Cohen’s kappa statistic was used to assess inter-rater agreement pairwise between the visual scoring assessment of the three readers (K.T, Sz.T. and K.B, indicated as assessor 1, two and three respectively throughout) for each of the investigated markers [25]. Spearman’s rank correlation was used to evaluate the association between the scoring values of experts (on the Likert scale 0–3) or the semiquantitatively segmented HistoQuant estimates (NGFR and Cx43) for each marker and grade respectively. Linear regression lines were fitted as orientation. The Kruskal-Wallis rank sum test was used as a global trend test to investigate the difference between L-NGFR, CXCL12, pERK12, Cx43 positivity and MF grades (MF-1, MF-2 and MF-3). As post hoc tests, Wilcoxon-Mann-Whitney U tests for two samples comparing their mean rank for each pair of grade levels were applied [25]. Figures were generated with the ggplot2 library using colorblind-friendly palettes. *p*-values were adjusted for multiple testing to counteract type 1 error inflation using the conservative Bonferroni correction (p*) [25]. Adjusted *p*-values <0.05 were considered significant.

## Results

### Expression of the Tested Markers in Non-fibrotic Bone Marrow

**FIGURE 1:**
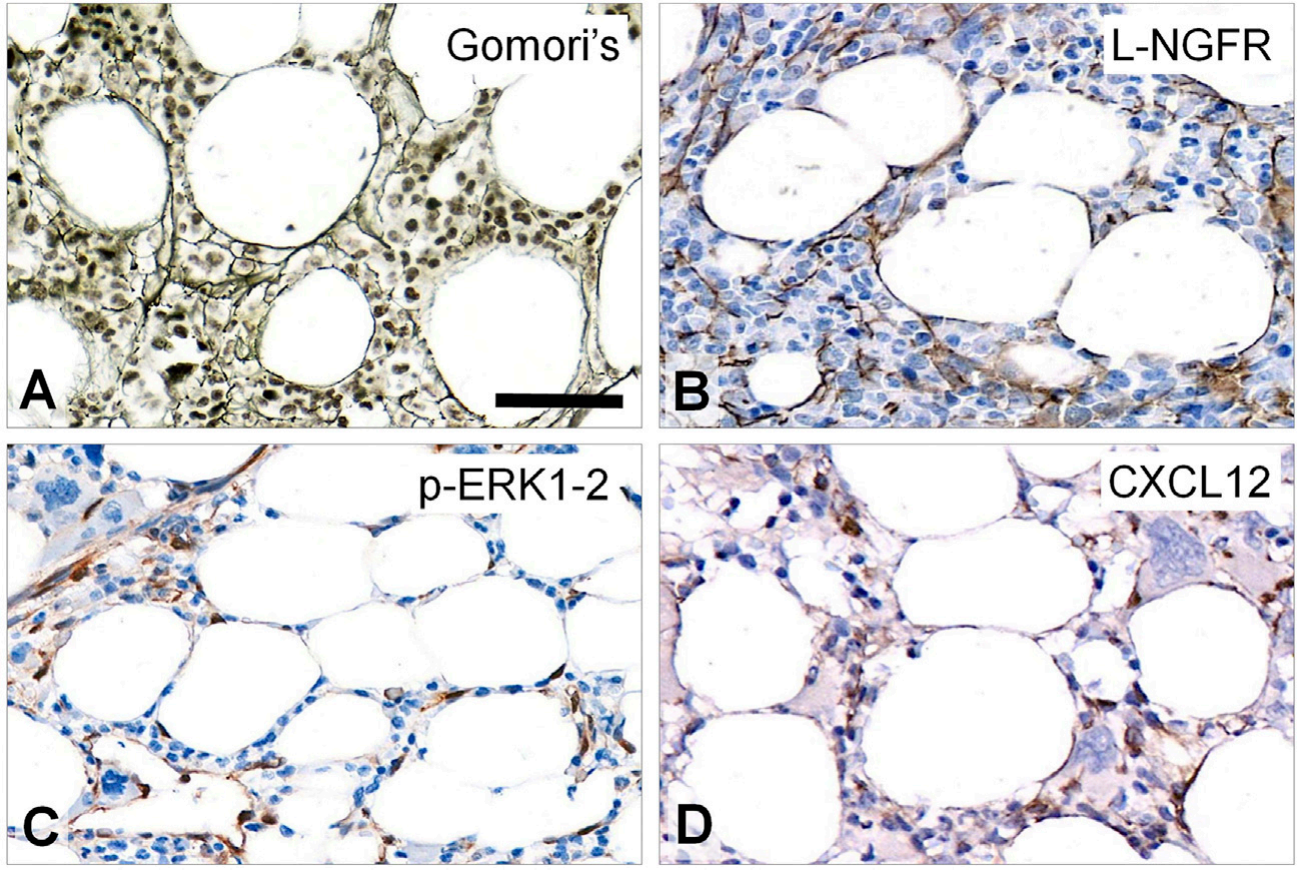
Staining pattern of the tested immunoreactions in intact areas of MF-1 bone marrow samples compared to Gomori’s silver impregnation **(A)**. L-NGFR highlights random stromal cells with their thin processes **(B)**. Phosphorylated ERK1-2 (*p*-ERK1-2) is detected mainly in oval nuclei of stromal and endothelial cells **(C)**, while CXCL12 reaction stains cell bodies of these elements **(D)** with both revealing only few cell processes. DAB immunoperoxidase reactions (brown) counterstained using hematoxylin **(B–D)**. Scale bar: 50 µm.

In normal-looking, intact areas of MF-1 bone marrow samples L-NGFR was detected in the bodies and processes of elongated, unevenly arranged stromal cells both in peri-trabecular and peri-sinusoidal regions with sporadic interconnections similar to Gomori’s silver impregnation (Figure 1A,B). Phosphorylated ERK1-2 was primarily seen in oval cell nuclei and rarely in their processes (Figure 1C), while CXCL12 positivity was linked to randomly dispersed, elongated cell bodies and scarce processes, along with a weak extracellular reaction (Figure 1D).

### Expression of the Tested Markers in Myelofibrosis

**FIGURE 2:**
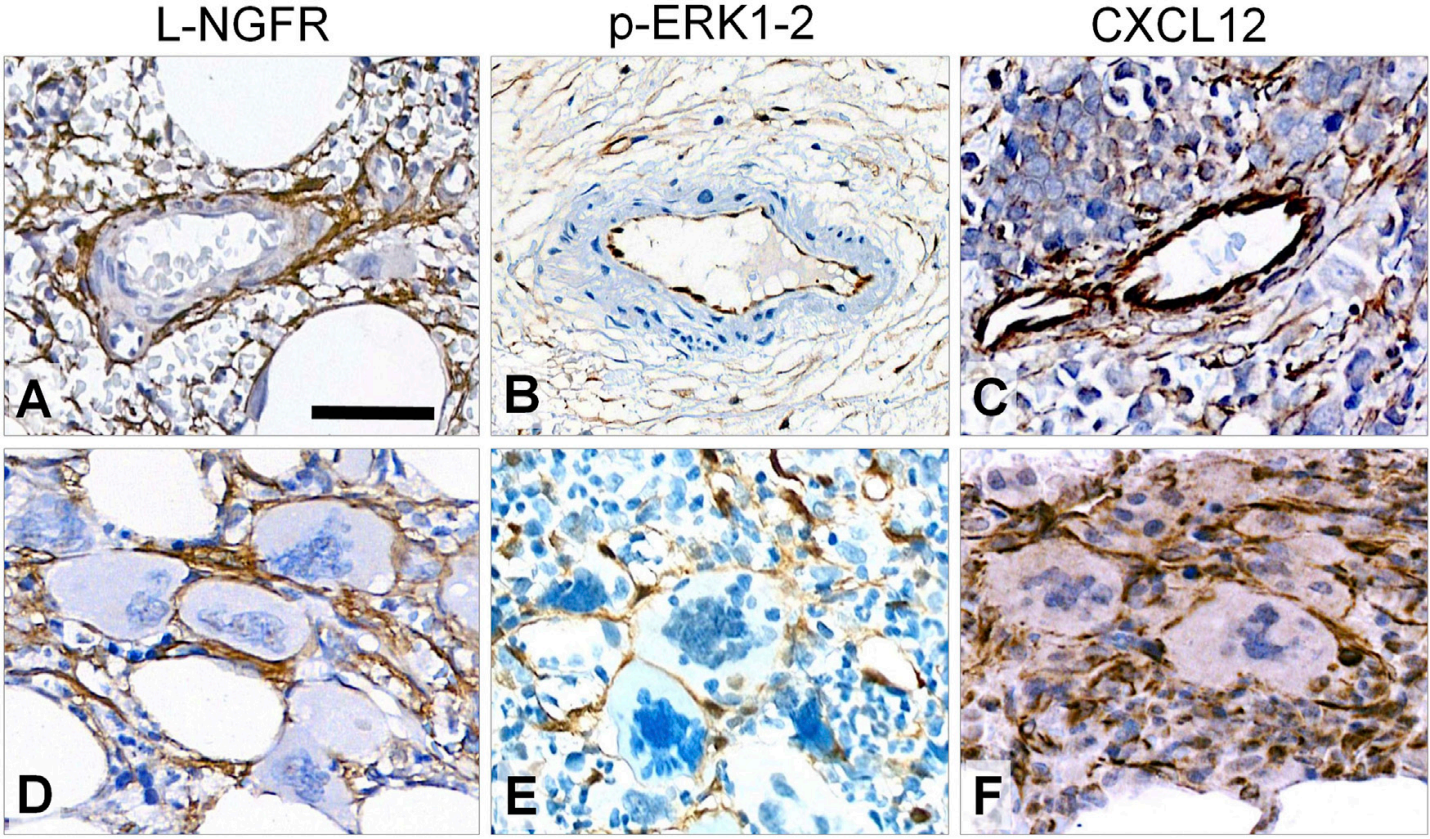
Immunoreaction patterns of the tested markers in arteries, periarterial stroma and abnormal megakaryocytes in myelofibrosis. L-NGFR positive stromal network spread from adventitial pericytes without endothelial reaction **(A)**; while both *p*-ERK1-2 **(B)** and CXCL12 **(C)** reactions occur in endothelial and inter-arteriolar stromal cells with rare interconnections (upper row). Immunopositive elongated cell processes are intimately associated with aberrant megakaryocytes with all three markers **(D, E** and **F**; lower panel**)**. DAB immunoperoxidase reactions (brown) counterstained using hematoxylin. Scale bar is 100 µm on A and B; and 50 µm on C-F.

All tested immunoreactions showed elevated number of marker positive cells in line with the increasing fibrosis. L-NGFR staining in perisinusoidal fibroblasts (pericytes) was obvious around arteri (oles)es, and high power view also confirmed this around capillary sinuses, but without any staining of the inner lining endothelial cells (Figure 2A). This was the only marker restricted to stromal cells, while both *p*-ERK1-2 and CXCL12 reactions were also seen in endothelial cells (Figure 2B,C). Immunopositive cell processes were frequently detected in close association with aberrant megakaryocytes with all three marker reactions (Figure 2D–F), suggesting a close interaction and functional cooperation with them.

### Correlations Between Growth Related Marker Expression and Myelofibrosis Grade

#### L-NGFR Scores as the Best Discriminators Among Myelofibrosis Grades

The incidence and density of stromal cell bodies and processes positive for the tested markers at the “hot spots” (where marker reactions were seen at the highest density), were compared to the Gomori’s-reticulin silver staining based grading using a similar 3-tier scale 1-2-3.

Immunoreactions were scored independently by three expert assessors and correlated with myelofibrosis grades both separately for each assessor and after consolidating the scores finally agreed on by all assessors.

**FIGURE 3:**
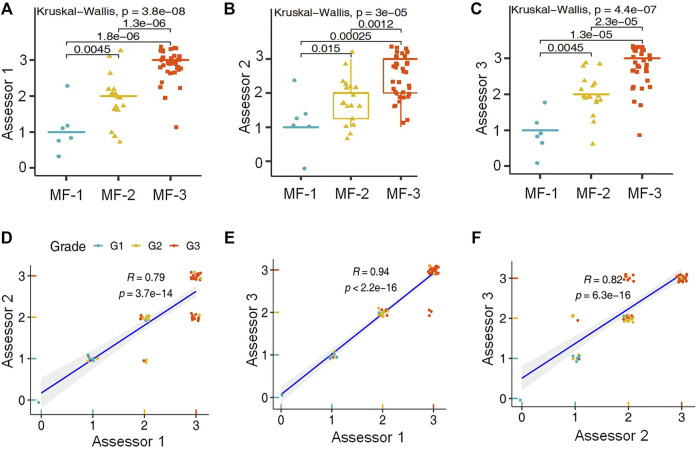
Statistical correlations between L-NGFR immunoreaction scores and Gomori’s silver staining based myelofibrosis grades as recognized by the individual assessors after using both the Kruskal-Wallis test and the Wilcoxon post-hoc test **(A–C)**. Pearson’s correlation values among scores of L-NGFR immunoreactions revealed between pairs of assessors in relation to Gomori’s silver staining based myelofibrosis grades **(D–F)**.

**FIGURE 4:**
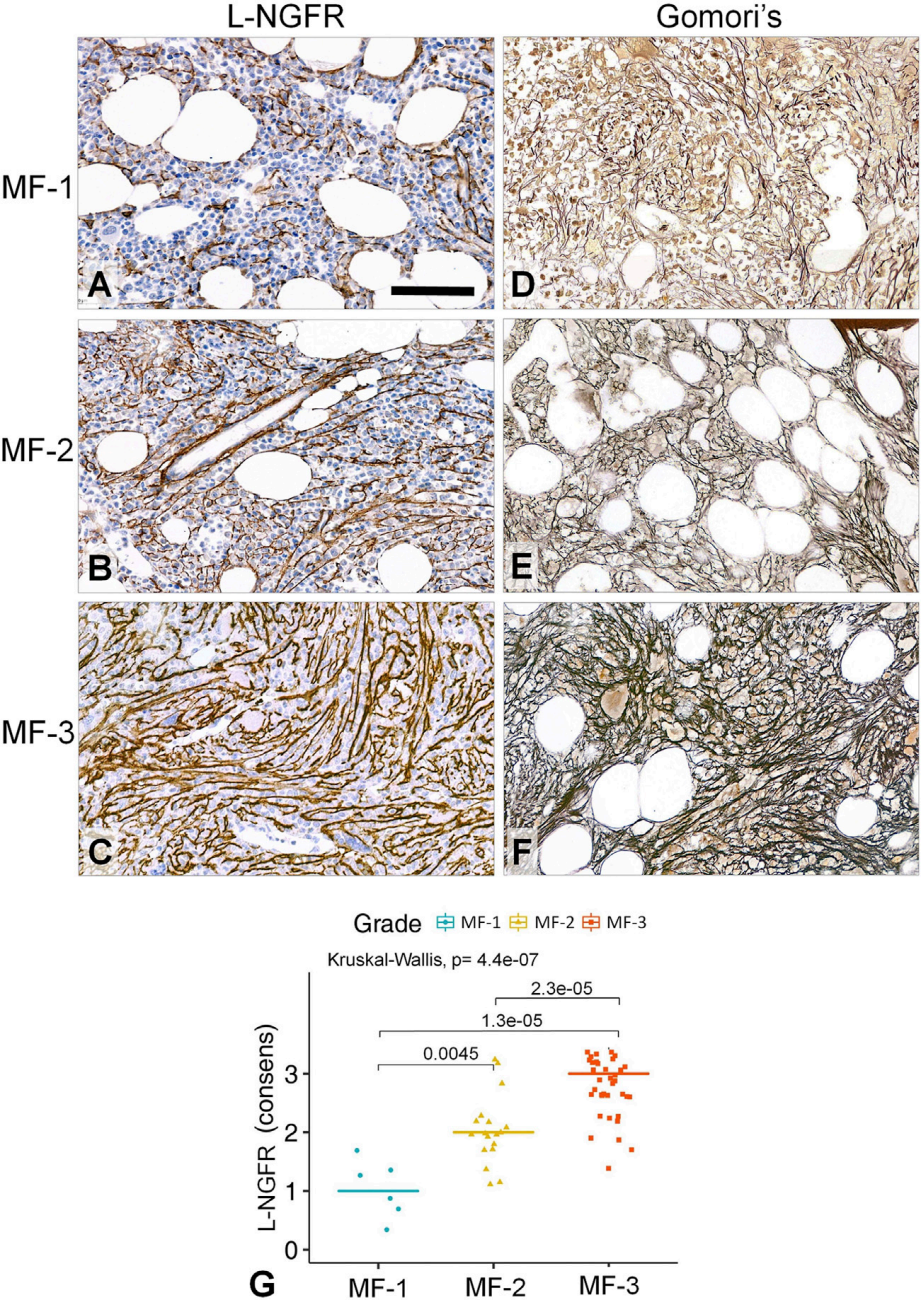
Correlations between the L-NGFR immunopositive stromal network **(A–C)** and the reticulin (and collagen) scaffolding **(D–F)** in myelofibrotic bone marrow samples. The gradually increasing density with some interconnections among L-NGFR positive cell processes show good correlation with reticular (and collagen) fibrosis determining myelofibrosis grade as revealed by Gomori’s silver impregnation. DAB immunoperoxidase reactions (brown) counterstained using hematoxylin **(A–C)**. Scale bar: 50 µm. High statistical correlation was detected between the consensus scores of the three assessors and the sliver impregnation based fibrosis grades both with the Kruskal-Wallis global trend test and the pairwise Wilcoxon post-hoc test **(G)**.

By using the Kruskal-Wallis rank sum test and the Wilcoxon-Mann-Whitney post-hoc test the scoring results of all three biomarker reactions proved to be suitable for differentiating myelofibrosis grades. L-NGFR positive stromal cell density and the related scores showed the highest statistical correlations with the Gormori’ silver staining based fibrosis grades at all three assessors (Figure 3A–C). Also, Spearman-rank analysis of L-NGFR scores showed high statistical link between assessors’ results when they were tested in pairs (Figure 3D–F). The progressively increasing density of L-NGFR positive stromal cell processes (Figure 4A–C) were matched with that of the reticular and collagen fibers of silver impregnation when demonstrated in parallel sections (Figure 4D–F). However, the less interconnections among L-NGFR positive projections compared to silver stained fibers was obvious. The consolidated immunoscores agreed on by all assessors resulted in Kuskall-Wallis *p* = 4.4e-07, along with *p* = 0.0045 between grade 1 and grade 2; *p* = 1.3e-05 between grade 1 and grade 3; and *p* = 2.3e-05 between grade 2 and grade 3 cases when using the Wilcoxon post-hoc test (Figure 4G). Cohen’s kappa values reflecting the inter-rater agreement between assessors scores on 60 cases also showed moderate to strong agreement. The association was moderate between assessors one and 2 (kappa = 0.682; z = 7.92; *p* = 1.41e-11); and between assessors two and 3 (kappa = 0.664; z = 7.41; *p* = 1.27e-13) and strong between assessors one and 3 (kappa = 0.916; z = 9.7; *p* = 0).

### Correlations of pERK or CXCL12 Scores With Myelofibrosis Grades

**FIGURE 5:**
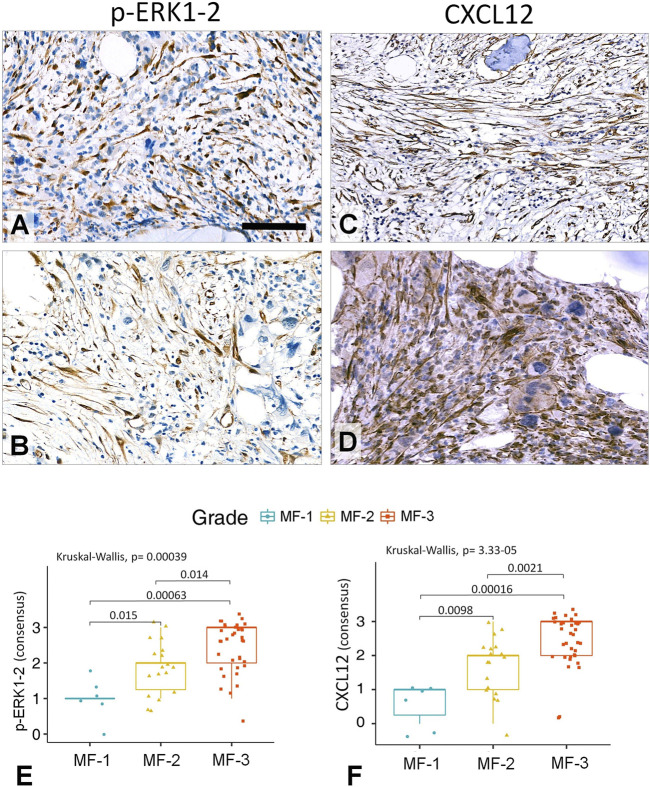
Representative examples of grade 3 myelofibrotic bone marrow samples where either *p*-ERK1-2 **(A,B)** or CXCL12 **(C,D)** immunoreactions reveal elongated stromal cells, which however, are rarely interconnected by their processes into contiguous meshworks. DAB immunoperoxidase reactions (brown) counterstained using hematoxylin. Scale bar is 50 µm on A, B and D; and 100 µm on **C**. Both the *p*-ERK1-2 **(E)** and CXCL12 **(F)** immunoreaction consensus scores showed statistical correlations with reticulin-silver staining based myelofibrosis grades either when using Kruskal-Wallis trend test or the pairwise Wilcoxon test. However, their discrimination power was weaker than that of L-NGFR.

Both *p*-ERK1-2 and CXCL12 scores showed less prominent significance concerning their grade discrimination values compared to that of L-NGFR. These markers occurred mainly in stromal cell nuclei (*p*-ERK1-2) and bodies (both *p*-ERK1-2 and CXCL12) projecting only thin processes, with almost no interconnections (Figure 5A–D). The consolidated immunoscores for *p*-ERK1-2 resulted in Kuskall-Wallis *p* = 0.00039, besides *p* = 0.015 between grade 1 and grade 2; *p* = 0.0063 between grade 1 and grade 3; and *p* = 0.014 between grade 2 and grade 3 cases when using the Wilcoxon post-hoc test. For CXCL12 the consolidated immunoscores led to Kruskal-Wallis *p* = 3.3e-05, followed by *p* = 0.0098 between grade 1 and grade 2; *p* = 0.00016 between grade 1 and grade 3; and *p* = 0.0021 between grade 2 and grade 3 cases when using the Wilcoxon post-hoc test. Both *p*-ERK1-2 and CXCL12 immunoreactions were less evident for straight assessment, which was also reflected by their weaker Spearman-rank correlations (*p*-ERK1-2, R = 0.76–0.81; CXCL12 R = 0.82–0.89) and Cohen’s kappa interrater values only weak correlations (*p*-ERK-1-2, kappa = 0.415–0.526; CXCL12, kappa = 0.512–0.578) than those of NGFR.

### Colocalization of L-NGFR Positive Stromal Network With Connexin 43 Gap Junction Plaques

**FIGURE 6:**
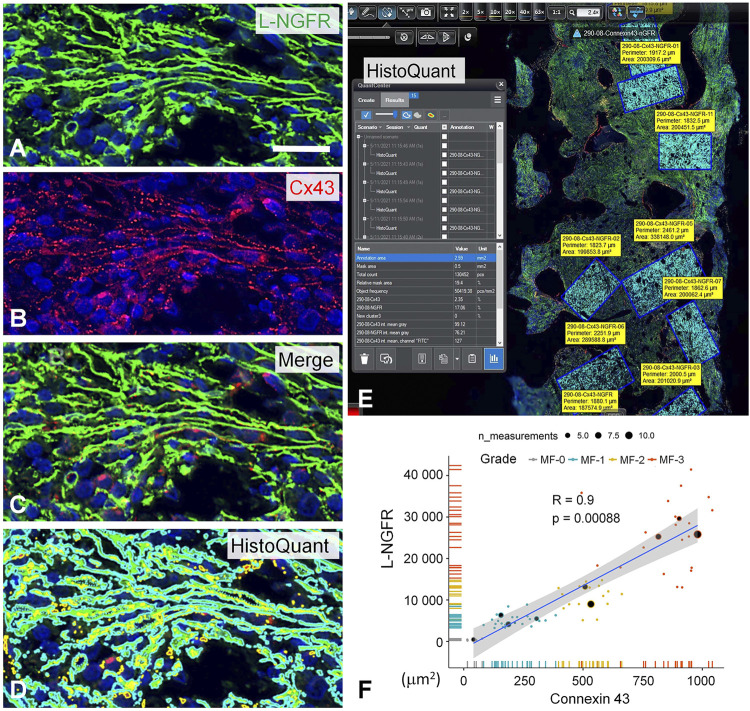
Double immunofluorescence for L-NGFR (green, **A)** and Cx43 (red, **B)** along with Hoechst nuclear counterstain (blue) in a grade 3 myelofibrosis. Merged images proved massive colocalization of the biomarkers **(C)**, which became apparent after highlighting the green (in turquoise) and red signals (merged in yellow) with the image segmentation algorithm of the HistoQuant software **(D)**. Semiautomated image analysis of the area fractions of fluorescent signals with this program in 67 standard areas of 10 myelofibrotic marrow samples **(E)** revealed strong overlap and correlation between L-NGFR and Cx43 signals suggesting that most particulate Cx43 belong to L-NGFR positive stromal cells **(F)**. Scale bar on A is 25 µm on A-D and 500 µm on E.

Earlier we observed that Cx43 direct cell-cell communication channels were upregulated in pathological bone marrow samples where stromal/hemopoetic cell ratio was increased [19]. Therefore, L-NGFR as the best marker to highlight stromal network in this study and Cx43 protein were detected simultaneously using immunofluorescence in samples representing all grades of myelofibrosis to see their potential link (Figure 6A–C). Multilayer scanning revealed Cx43 positive particles of <1 micrometer throughout the ∼4 micrometer section thickness and their significant co-localization with L-NGFR positive stromal cell processes. The HistoQuant software was used for quantifying both reactions in representative areas (67 areas of 10 cases) after image segmentation highlighting the specific reactions in each channel (Figure 6D,E). The massive coexpression of the two biomarker reactions was obvious after merging the segmented immune signals (Figure 6D). Spearman’s rank test was used to evaluate the association between the expression of the two makers, which showed strong statistical correlations (Figure 6E).

## Discussion

Despite the novel prognostic models considering driver and passenger mutations, karyotype and sex-adjusted hemoglobin levels, bone marrow fibrosis reflecting the abnormal activation of the fibrotic stromal microenvironment is still a major predictor of the outcome in primary myelofibrosis [2, 5]. Here we tested growth related markers which were primarily expressed by stromal cells including L-NGFR/CD271, *p*-ERK1-2 and CXCL-12, and found that the density of positive cells for each marker was progressively increased in line with the Gomori’s silver staining-based tumor grades. NGFR reactions restricted to and revealed the whole length of stromal cells (the others also showed some endothelial positivity) with most intersections of the 3 markers, showed the best statistical correlation and the most reproducible agreement among assessors’ judgements with myelofibrosis grades. L-NGFR positive stromal cells co-expressed Cx43 protein, particularly in advanced myelofibrosis, suggesting an enhanced communication and functional coupling within the overgrown stromal network. Therefore, our findings revealed some biomarkers of stromal cell activation of which L-NGFR expression proved to be the most promising to complement Gomori’s silver staining for myelofibrosis grading.

Myelofibrosis is driven by augmented and pathological extracellular matrix production resulted from the increasing number and activation of bone marrow stromal cells as a result of aberrant cytokine signaling and clonal megakaryocyte functions [3]. The extent of matrix overproduction, still assessed using Gomori’s silver impregnation, determines grades in primary myelofibrosis and predicts disease progression and outcome [2]. However, selective silver deposition on reticular and collagen fibers can be highly dependent on preanalytical (fixation, decalcification) and staining (components, timing, ambient temperature and light conditions) variables [5]. Furthermore, it can’t directly reveal the functional activation of stromal cells and differentiate between “prefibrotic” and “overtly fibrotic” phase of primary myelofibrosis [1]. Though none of our selected markers highlighted the reticular or collagen matrix in the bone marrow, they were primarily expressed in stromal cells and were linked to growth related pathways as potential indicators of their activation. Since MF-0, “normal-looking bone marrow” showed only few spindle shaped positive stromal cells in random localization when the immunoreactions were set up, very similar to the intact looking areas in MF-1 cases (see Figure 1), we considered these to represent the basic MF-0 situation. Intimate association of spindle shaped stromal cells, which were positive for each marker, with aberrant megakaryocytes was likely to reflect their functional co-operation and the potential impact of cytokines and growth factors released by megakaryocytes on pathological stromal cell activation.

L-NGFR (CD271) is a low affinity receptor for neurotrophins including NGF, which by cooperating with tropomyosin receptor kinase A (TrkA) can catalyze its receptor tyrosine kinase (RTK) mediated signaling in neurons [26, 27]. Alternatively, L-NGFR may prevent this growth signaling by interacting with non-preferred neurotrophins, which may lead to programmed cell death response (apoptosis) e.g. in oligodendrocytes [28]. In our study, L-NGFR was progressively expressed in the growing stromal cell network along with disease progression in myelofibrosis, which suggests its involvement in growth promotion rather than in apoptosis. TrkA may assist L-NGFR in this, since both of them are thought to be involved in mesenchymal stromal cell functions [29], despite that Trk neurotrophin receptors have been only rarely detected in bone marrow stromal network [30]. Nevertheless, the finding that upregulated TrkA in bone marrow stromal stem cells can augment their survival and regenerative capacity in nerve grafts through the MAPK pathway, can also support this view [11]. Bone marrow stromal cells may also produce nerve growth factor which raises a potential autocrine regulation of stromal proliferation too [31].

L-NGFR expression was first observed in bone marrow stromal cells nearly 30 years ago, when its correlation with reticulin silver staining in myelofibrosis was already mentioned, but it was based on only 2 cases [6]. Immuno-electron microscopy of L-NFGR confirmed labeling all along stellate or spindle-shaped filamentous stromal processes, primarily in bone marrow adventitial reticular cells [32]. This supports our finding of L-NGFR immunoreaction through the whole length of stromal cell processes in the myelofibrotic bone marrow. L-NGFR/CD271 has been considered as a selective marker for mesenchymal stromal (stem) cell isolation, since positive cell fraction were rich in clonogenic precursors, which showed increased proliferation [33] and trilineage differentiation into fibroblastic, adipocytic and osteoblastic cells, significantly more than CD271 negative stromal cells [9, 34]. These findings also suggest that L-NGFR/CD271 is involved in the functional activation of bone marrow stromal network. Though L-NGFR immunoreaction was recommended for differential diagnosis within myeloproliferative neoplasms by showing that positive stromal cells were more frequent in primary myelofibrosis that in essential thrombocythemia (ET) or in polycythaemia vera (PV) [35], its correlations with myelofibrosis grades had not been studied systematically before. In our work, L-NGFR reaction showed the strongest discriminating power of the tested biomarkers among myelofibrosis grades as shown both by the Kruskal-Wallis global trend-tests and the Wilcoxon post-hoc test. This was the only marker to be detected exclusively in stromal cells and throughout their processes. Furthermore, L-NGFR scores demonstrated the best interrater agreement between pairs of assessors and thus L-NGFR immunoreaction can be a biological indicator of stromal cell activation and overproduction of reticular and collagen fibers revealed by silver impregnation.

Receptor tyrosine kinase (RTK) signaling is the major promoter of profibrogenic responses [36]. RTKs (TrkA may be too) have been strongly involved in the regulation of mesenchymal stromal cell activation, growth and proliferation, including the bone marrow stromal microenvironment, mainly through PDGFR, EGFR [37] and FGF2/bFGF receptors [38]. PDGFR-β (but not PDGFR-⟨) expression was found to indicate the functional activation of bone stromal cells in the myelofibrotic marrow and to statistically correlate with the Gomori’s silver impregnation based fibrosis grades [13-15]. RTK signaling converges to ERK1-2 phosphorylation as a sign of its activation, resulting in its translocation into cell nuclei to promote growth and proliferation related adaptation responses in stromal fibroblasts as a transcription factor [39, 40]. In agreement with this, our *p*-ERK1-2 immunoreactions occurred both in the nuclei and the cytoplasm of spindle shaped cells. However, the reaction did not highlight the whole length of stromal cell processes, thus rarely showed interconnections, and also occurred in some sinus endothelial cells of similar morphology. Despite these limitations, *p*-ERK1-2 protein positive cell density statistically correlated with myelofibrosis progression in line with Gomori’s silver impregnation. Though *p*-ERK1-2 immunoreaction proved to be another biomarker of bone marrow stromal cell activation and myelofibrosis evolution it showed less potential than NGFR reaction for diagnostic use.

The CXCL12 chemokine is constitutively released from bone marrow stromal cells and contributes to maintaining stem cells in bone marrow niches by binding to CXCR4 on CD34+/CD117 + hemopoietic progenitors and leukemic blasts too [16]. Therefore, CXCL12 may also be involved in protecting the leukemic stem cells and contributing to myelofibrosis progression [22], though CXCR4 is downregulated in the malignant progenitors, which may explain extramedullary clonal hematopoiesis in myelofibrosis [41]. Endothelial cells may also express CXCL12, although at a lower level [16]. CXCL12 plays an essential role in stem cell homing at transplantation and the CXCL12/CXCR4 interaction supports stromal cell resistance and restoration of the stromal network after myeloablative irradiation [17]. CXCL12/CXCR4 pathway is activated by oncogenic JAK2, which frequently suffers activating mutations in primary myelofibrosis [20]. In line with this, we detected CXCL12 positive perivascular and peri‐osteoblastic stromal cells at increasing density with the Gomori’s silver staining based myelofibrosis grades. However, similar to that of *p*-ERK1-2, CXCL12 immunoreactions might not reveal all stromal cells and highlight only part of stromal cell bodies. The mild extracellular staining was in line with CXCL12 functions, which, however, is another reason preventing it to be a useful diagnostic biomarker of myelofibrosis progression.

Connexins form direct cell-cell communication channels that allow the controlled passage of small (<1.8 kDa) regulatory molecules between coupled cells and thus, support the formation of functional cell compartments within tissues including the bone marrow [19]. CXCL12 release requires close cooperation of stromal cells, which need to function as a network coupled by Cx43 (and less by CX45) gap junctions [18]. In line with our earlier study [19], we confirmed this close association in the form of protein colocalization between the stromal L-NGFR immunoreaction and Cx43 gap junction plaques. Their parallel and progressively elevated expression suggests an increasing functional activation and potential communication within the stromal network in myelofibrosis in line with disease advancement.

In conclusion, all of our tested growth related biomarkers including L-NGFR, *p*-ERK1-2 and CXCL12 were likely to be expressed in activated bone marrow stromal cells and their immunoscores were statistically correlated with Gomori’s silver impregnation based myelofibrosis grades at varying strength. L-NGFR reaction was restricted to and revealed the whole length of stromal cells, and showed the best correlations with myelofibrosis grades and the strongest interrater agreements. The progressive expression of Cx43 gap junctions in the stromal cells may support compartmental co-ordination within the myelofibrotic stroma. Our results suggest that L-NGFR expression can be a useful biological marker of stromal cell activation which deserves diagnostic consideration for complementing Gomori’s silver impregnation. Further, direct biological evidence of stromal activation will be gained by detecting the expression of different matrix proteins in myelofibrosis, which is under way in our laboratory.

## Data Availability

The raw data supporting the conclusions of this article will be made available by the authors, without undue reservation.

## References

[1] Barbui T Thiele J Gisslinger H Kvasnicka HM Vannucchi AM Guglielmelli P The 2016 WHO Classification and Diagnostic Criteria for Myeloproliferative Neoplasms: Document Summary and In-Depth Discussion. Blood Cancer J (2018) 8:15. 10.1038/s41408-018-0054-y 29426921 PMC5807384

[2] Tefferi A. Primary Myelofibrosis: 2019 Update on Diagnosis, Risk-Stratification and Management. Am J Hematol (2018) 93:1551–60. 10.1002/ajh.25230 30039550

[3] Schieber M Crispino JD Stein B. Myelofibrosis in 2019: Moving beyond JAK2 Inhibition. Blood Cancer J (2019) 9:74. 10.1038/s41408-019-0236-2 31511492 PMC6739355

[4] Gömöri G. Silver Impregnation of Reticulum in Paraffin Sections. Am J Pathol (1937) 13:993–5. 19970363 PMC1911151

[5] Kvasnicka HM Beham‐Schmid C Bob R Dirnhofer S Hussein K Kreipe H Problems and Pitfalls in Grading of Bone Marrow Fibrosis, Collagen Deposition and Osteosclerosis - a Consensus‐based Study. Histopathology (2016) 68:905–15. 10.1111/his.12871 26402166

[6] Cattoretti G Schiró R Orazi A Soligo D Colombo M. Bone Marrow Stroma in Humans: Anti-nerve Growth Factor Receptor Antibodies Selectively Stain Reticular Cells *In Vivo* and *In Vitro* . Blood (1993) 81:1726–38. 10.1182/blood.v81.7.1726.bloodjournal8171726 7681701

[7] Covaceuszach S Konarev PV Cassetta A Paoletti F Svergun DI Lamba D The Conundrum of the High-Affinity NGF Binding Site Formation Unveiled? Biophysical J (2015) 108:687–97. 10.1016/j.bpj.2014.11.3485 PMC4317559 25650935

[8] Micera A Lambiase A Stampachiacchiere B Bonini S Bonini S Levischaffer F. Nerve Growth Factor and Tissue Repair Remodeling: trkANGFR and p75NTR, Two Receptors One Fate. Cytokine Growth Factor Rev (2007) 18:245–56. 10.1016/j.cytogfr.2007.04.004 17531524

[9] Barilani M Banfi F Sironi S Ragni E Guillaumin S Polveraccio F Low-affinity Nerve Growth Factor Receptor (CD271) Heterogeneous Expression in Adult and Fetal Mesenchymal Stromal Cells. Sci Rep (2018) 8:9321. 10.1038/s41598-018-27587-8 29915318 PMC6006357

[10] Tomlinson RE Li Z Zhang Q Goh BC Li Z Thorek DLJ NGF-TrkA Signaling by Sensory Nerves Coordinates the Vascularization and Ossification of Developing Endochondral Bone. Cel Rep (2016) 16:2723–35. 10.1016/j.celrep.2016.08.002 PMC5014649 27568565

[11] Zheng MG Sui WY He ZD Liu Y Huang YL Mu SH TrkA Regulates the Regenerative Capacity of Bone Marrow Stromal Stem Cells in Nerve Grafts. Neural Regen Res (2019) 14:1765–71. 10.4103/1673-5374.257540 31169194 PMC6585565

[12] Bock O Loch G Büsche G von Wasielewski R Schlué J Kreipe H. Aberrant Expression of Platelet-Derived Growth Factor (PDGF) and PDGF Receptor-Alpha Is Associated with Advanced Bone Marrow Fibrosis in Idiopathic Myelofibrosis. Haematologica (2005) 90:133–4. 15642683

[13] Bedekovics J Kiss A Beke L Károlyi K Méhes G. Platelet Derived Growth Factor Receptor-Beta (PDGFRβ) Expression Is Limited to Activated Stromal Cells in the Bone Marrow and Shows a strong Correlation with the Grade of Myelofibrosis. Virchows Arch (2013) 463:57–65. 10.1007/s00428-013-1434-0 23748876

[14] Bedekovics J Szeghalmy S Beke L Fazekas A Méhes G. Image Analysis of Platelet Derived Growth Factor Receptor-Beta (PDGFRβ) Expression to Determine the Grade and Dynamics of Myelofibrosis in Bone Marrow Biopsy Samples. Cytometry (2014) 86:319–28. 10.1002/cyto.b.21167 24810671

[15] Méhes G Tzankov A Hebeda K Anagnostopoulos I Krenács L Bedekovics J. Platelet-derived Growth Factor Receptor β (PDGFRβ) Immunohistochemistry Highlights Activated Bone Marrow Stroma and Is Potentially Predictive for Fibrosis Progression in Prefibrotic Myeloproliferative Neoplasia. Histopathology (2015) 67:617–24. 10.1111/his.12704 25825163

[16] Kitagawa M Kurata M Onishi I Yamamoto K. Bone Marrow Niches in Myeloid Neoplasms. Pathol Int (2020) 70:63–71. 10.1111/pin.12870 31709722 PMC7232432

[17] Singh P Mohammad KS Pelus LM. CXCR4 Expression in the Bone Marrow Microenvironment Is Required for Hematopoietic Stem and Progenitor Cell Maintenance and Early Hematopoietic Regeneration after Myeloablation. Stem cells (Dayton, Ohio) (2020) 38:849–59. 10.1002/stem.3174 PMC7566894 32159901

[18] Schajnovitz A Itkin T D'Uva G Kalinkovich A Golan K Ludin A CXCL12 Secretion by Bone Marrow Stromal Cells Is Dependent on Cell Contact and Mediated by Connexin-43 and Connexin-45 gap Junctions. Nat Immunol (2011) 12:391–8. 10.1038/ni.2017 21441933

[19] Krenacs T Rosendaal M. Connexin43 gap Junctions in normal, Regenerating, and Cultured Mouse Bone Marrow and in Human Leukemias: Their Possible Involvement in Blood Formation. Am J Pathol (1998) 152:993–1004. 9546360 PMC1858239

[20] Abdelouahab H Zhang Y Wittner M Oishi S Fujii N Besancenot R CXCL12/CXCR4 Pathway Is Activated by Oncogenic JAK2 in a PI3K-dependent Manner. Oncotarget (2017) 8:54082–95. 10.18632/oncotarget.10789 28903325 PMC5589564

[21] López-Gil JC Martin-Hijano L Hermann PC Sainz B Jr. The CXCL12 Crossroads in Cancer Stem Cells and Their Niche. Cancers (Basel) (2021) 13. 10.3390/cancers13030469 PMC7866198 33530455

[22] Schepers K Pietras EM Reynaud D Flach J Binnewies M Garg T Myeloproliferative Neoplasia Remodels the Endosteal Bone Marrow Niche into a Self-Reinforcing Leukemic Niche. Cell stem cell (2013) 13:285–99. 10.1016/j.stem.2013.06.009 23850243 PMC3769504

[23] Thiele J Kvasnicka HM Facchetti F Franco V van der Walt J Orazi A. European Consensus on Grading Bone Marrow Fibrosis and Assessment of Cellularity. Haematologica (2005) 90:1128–32. 16079113

[24] Balla P Maros ME Barna G Antal I Papp G Sapi Z Prognostic Impact of Reduced Connexin43 Expression and gap junction Coupling of Neoplastic Stromal Cells in Giant Cell Tumor of Bone. PloS one (2015) 10:e0125316. 10.1371/journal.pone.0125316 25933380 PMC4416750

[25] Maros ME Wenz R Förster A Froelich MF Groden C Sommer WH Objective Comparison Using Guideline-Based Query of Conventional Radiological Reports and Structured Reports. In Vivo (2018) 32:843–9. 10.21873/invivo.11318 29936469 PMC6117779

[26] Canossa M Twiss JL Verity AN Shooter EM. p75(NGFR) and TrkA Receptors Collaborate to Rapidly Activate a p75(NGFR)-associated Protein Kinase. EMBO J (1996) 15:3369–76. 10.1002/j.1460-2075.1996.tb00702.x 8698038 PMC451900

[27] Barker PA. p75NTR: A Study in Contrasts. Cell Death Differ (1998) 5:346–56. 10.1038/sj.cdd.4400375 10200483

[28] Casaccia-Bonnefil P Kong H Chao MV. Neurotrophins: the Biological Paradox of Survival Factors Eliciting Apoptosis. Cel Death Differ (1998) 5:357–64. 10.1038/sj.cdd.4400377 10200484

[29] Zha K Yang Y Tian G Sun Z Yang Z Li X Nerve Growth Factor (NGF) and NGF Receptors in Mesenchymal Stem/stromal Cells: Impact on Potential Therapies. Stem Cell translational Med (2021) 10:1008–20. 10.1002/sctm.20-0290 PMC8235142 33586908

[30] Labouyrie E Dubus P Groppi A Mahon FX Ferrer J Parrens M Expression of Neurotrophins and Their Receptors in Human Bone Marrow. Am J Pathol (1999) 154:405–15. 10.1016/s0002-9440(10)65287-x 10027399 PMC1849993

[31] García R Aguiar J Alberti E de la Cuétara K Pavón N. Bone Marrow Stromal Cells Produce Nerve Growth Factor and Glial Cell Line-Derived Neurotrophic Factors. Biochem Biophys Res Commun (2004) 316:753–4. 10.1016/j.bbrc.2004.02.111 15033464

[32] Caneva L Soligo D Cattoretti G De Harven E Lambertenghi Deliliers G. Immuno-electron Microscopy Characterization of Human Bone Marrow Stromal Cells with Anti-NGFR Antibodies. Blood Cell Mol Dis (1995) 21:73–85. 10.1006/bcmd.1995.0011 8846047

[33] Calabrese G Giuffrida R Lo Furno D Parrinello N Forte S Gulino R Potential Effect of CD271 on Human Mesenchymal Stromal Cell Proliferation and Differentiation. Ijms (2015) 16:15609–24. 10.3390/ijms160715609 26184166 PMC4519916

[34] Quirici N Soligo D Bossolasco P Servida F Lumini C Deliliers GL. Isolation of Bone Marrow Mesenchymal Stem Cells by Anti-nerve Growth Factor Receptor Antibodies. Exp Hematol (2002) 30:783–91. 10.1016/s0301-472x(02)00812-3 12135677

[35] Yigit N Covey S Barouk-Fox S Turker T Geyer JT Orazi A. Nuclear Factor-Erythroid 2, Nerve Growth Factor Receptor, and CD34-Microvessel Density Are Differentially Expressed in Primary Myelofibrosis, Polycythemia Vera, and Essential Thrombocythemia. Hum Pathol (2015) 46:1217–25. 10.1016/j.humpath.2015.05.004 26093937

[36] Beyer C Distler JHW. Tyrosine Kinase Signaling in Fibrotic Disorders. Biochim Biophys Acta (Bba) - Mol Basis Dis (2013) 1832:897–904. 10.1016/j.bbadis.2012.06.008 22728287

[37] Satomura K Derubeis AR Fedarko NS Ibaraki-O'Connor K Kuznetsov SA Rowe DW Receptor Tyrosine Kinase Expression in Human Bone Marrow Stromal Cells. J Cel Physiol. (1998) 177:426–38. 10.1002/(sici)1097-4652(199812)177:3<426::aid-jcp6>3.0.co;2-f 9808151

[38] Dupree MA Pollack SR Levine EM Laurencin CT. Fibroblast Growth Factor 2 Induced Proliferation in Osteoblasts and Bone Marrow Stromal Cells: a Whole Cell Model. Biophysical J (2006) 91:3097–112. 10.1529/biophysj.106.087098 PMC1578487 16861274

[39] Pagès G Lenormand P L'Allemain G Chambard JC Meloche S Pouysségur J. Mitogen-activated Protein Kinases P42mapk and P44mapk Are Required for Fibroblast Proliferation. Proc Natl Acad Sci (1993) 90:8319–23. 10.1073/pnas.90.18.8319 8397401 PMC47347

[40] Plotnikov A Zehorai E Procaccia S Seger R. The MAPK Cascades: Signaling Components, Nuclear Roles and Mechanisms of Nuclear Translocation. Biochim Biophys Acta (Bba) - Mol Cel Res (2011) 1813:1619–33. 10.1016/j.bbamcr.2010.12.012 21167873

[41] Song M-K Park B-B Uhm J-E. Understanding Splenomegaly in Myelofibrosis: Association with Molecular Pathogenesis. Ijms (2018) 19:898. 10.3390/ijms19030898 PMC5877759 29562644

